# Benchmark Dose for Cadmium-Induced Renal Effects in Humans

**DOI:** 10.1289/ehp.9028

**Published:** 2006-04-18

**Authors:** Yasushi Suwazono, Salomon Sand, Marie Vahter, Agneta Falk Filipsson, Staffan Skerfving, Jonas Lidfeldt, Agneta Åkesson

**Affiliations:** 1 Institute of Environmental Medicine, Karolinska Institutet, Stockholm, Sweden; 2 Department of Occupational and Environmental Medicine, Graduate School of Medicine, Chiba University, Chiba, Japan; 3 Swedish Chemicals Inspectorate, Sundbyberg, Sweden; 4 Department of Occupational and Environmental Medicine, Lund University, Lund, Sweden; 5 Department of Community Health, Malmö University Hospital, Malmö, Sweden

**Keywords:** benchmark dose, continuous data, environmental exposure, human, renal glomerular dysfunction, renal tubular dysfunction, risk assessment, urinary cadmium

## Abstract

**Objectives:**

Our goal in this study was to explore the use of a hybrid approach to calculate benchmark doses (BMDs) and their 95% lower confidence bounds (BMDLs) for renal effects of cadmium in a population with low environmental exposure.

**Methods:**

Morning urine and blood samples were collected from 820 Swedish women 53–64 years of age. We measured urinary cadmium (U-Cd) and tubular effect markers [*N*-acetyl- β-D-glucosaminidase (NAG) and human complex-forming protein (protein HC)] in 790 women and estimated glomerular filtration rate (GFR; based on serum cystatin C) in 700 women. Age, body mass index, use of nonsteroidal anti-inflammatory drugs, and blood lead levels were used as covariates for estimated GFR. BMDs/BMDLs corresponding to an additional risk (benchmark response) of 5 or 10% were calculated (the background risk at zero exposure was set to 5%). The results were compared with the estimated critical concentrations obtained by applying logistic models used in previous studies on the present data.

**Results:**

For both NAG and protein HC, the BMDs (BMDLs) of U-Cd were 0.5–1.1 (0.4–0.8) μg/L (adjusted for specific gravity of 1.015 g/mL) and 0.6–1.1 (0.5–0.8) μg/g creatinine. For estimated GFR, the BMDs (BMDLs) were 0.8–1.3 (0.5–0.9) μg/L adjusted for specific gravity and 1.1–1.8 (0.7–1.2) μg/g creatinine.

**Conclusion:**

The obtained benchmark doses of U-Cd were lower than the critical concentrations previously reported. The critical dose level for glomerular effects was only slightly higher than that for tubular effects. We suggest that the hybrid approach is more appropriate for estimation of the critical U-Cd concentration, because the choice of cutoff values in logistic models largely influenced the obtained critical U-Cd.

People are exposed to cadmium—a widespread nephrotoxic pollutant—via food and tobacco smoking. The first sign of renal effects is tubular damage, characterized by increased urinary excretion of low-molecular-weight proteins or intracellular tubular enzymes. More important, in succession to the tubular effects, Cd may affect glomerular function (Åkesson et al. 2005; Bernard et al. 1992; Friberg 1950; Järup et al. 1995; Nogawa 1984; World Health Organization 1992). To protect people from Cd-induced health effects, it is crucial to determine the critical exposure, that is, the concentration of urinary Cd (U-Cd) below which the probability of adverse health effects is low. Attempts to estimate this limit for tubular effects have so far displayed large variations in critical U-Cd levels (1–10 μg U-Cd/g creatinine) (Buchet et al. 1980, 1990; Hong et al. 2004; Järup et al. 2000; Jin et al. 2004; Lauwerys et al. 1979).

The benchmark dose (BMD) method is increasingly used in the health risk assessment of environmental contaminants [Crump 1984; Filipsson et al. 2003; U.S. Environmental Protection Agency (EPA) 1995]. Only in a few cases has the BMD method been used for people environmentally exposed to Cd (Hong et al. 2004; Jin et al. 2004; Uno et al. 2005). The BMD can be defined as the exposure that corresponds to a certain change in response compared with the background. The lower 95% confidence bound of the BMD (BMDL) has been suggested to replace the no observed adverse effect level (NOAEL) (Crump 1984; U.S. EPA 1995). One major advantage of the BMD/BMDL approach is that it uses the whole dose–response curve (U.S. EPA 1995). Thus, the BMD/BMDL is based on more information than the NOAEL. By using a so-called hybrid approach, the concept of risk can be used for a continuous outcome (effect variable). In that way, the limitations associated with categorization of data can be avoided (Crump 1995; Gaylor and Slikker 1990; Kodell and West 1993; Ragland 1992).

Our aim in the present study was to determine the BMDs of U-Cd for Cd-induced tubular and glomerular effects in an environmentally exposed population, using the hybrid approach. To evaluate the unique feature of the hybrid approach, the obtained BMDs/BMDLs were compared with the critical concentrations obtained by the traditionally used procedures.

## Materials and Methods

### Study population and measurement

Within the population-based Women’s Health in the Lund Area (WHILA) study (Lidfeldt et al. 2001), conducted in an area with no particular industrial emission, we assessed health effects of Cd in 820 women 53–64 years of age (Åkesson et al. 2005). Subjects with renal cancer and lithium treatment were excluded (*n* = 4). In addition, because of effect modification (Åkesson et al. 2005), insulin-treated subjects with diabetes were excluded from calculation of the BMD for tubular (*n* = 14) but not glomerular effects.

According to a questionnaire, 45% of the included women had ever smoked (ever-smokers). In addition, nonsteroidal anti-inflammatory drugs (NSAIDs) were regularly used by 6% of the women.

We used U-Cd as the measure of long-term Cd exposure, urinary *N*-acetyl-β-d-glu-cosaminidase (NAG) and human complex-forming protein (protein HC) as markers of tubular effects, and estimated glomerular filtration rate (GFR) based on cystatin C in serum (estimated GFR = 77.24 × cystatin C^–1.2623^) (Åkesson et al. 2005; Larsson et al. 2004) as a marker of glomerular effect (Table 1). Urinary analytes were adjusted to a specific gravity of 1.015 g/mL, because creatinine may not adjust for all dilution-related variation of U-Cd (Suwazono et al. 2005). However, because creatinine adjustment is more commonly used, these values are given for comparison. The ethics committee at Lund University approved the WHILA study, and oral informed consent was obtained from each participant.

### Model fitting

We used the maximum likelihood approach to fit the dose–response curve to the data (Crump 1995). For normally distributed data with constant variance, the log-likelihood function, log *L*, is given by


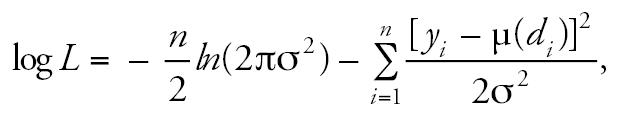


where *n* is the number of subjects, d*_i_* is the dose for *i*th individual, σ^2^ is the variance, μ (*d_i_*) is the dose–response model for the mean response, and *y_i_* is the response in the *i*th individual. To obtain a symmetrical distribution, data on NAG and protein HC were log-transformed. Data on estimated GFR did not have to be log-transformed. The model for the mean response, μ(*d_i_*), was assumed to be linear:


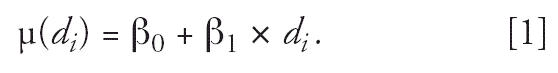


We found significant covariates only for estimated GFR (Åkesson et al. 2005). Age, body mass index, use of NSAIDs, and blood lead levels (Åkesson et al. 2005) were included in the model to control for possible confounding of estimated GFR; NAG and protein HC displayed no such associations. Smoking (pack-years) was not associated with any of the kidney effect markers.

### Calculation of BMDs

BMDs were calculated using a hybrid approach, which allows for calculation of risk for continuous data without dichotomizing the outcome (Crump 2002; Gaylor and Slikker 1990; Sand et al. 2004). The benchmark response (BMR), corresponding to the BMD, was defined as an additional prespecified increase in the probability of adverse response. For positive associations between exposure (U-Cd) and effects (NAG and protein HC), the effect level associated with a certain BMR equals.


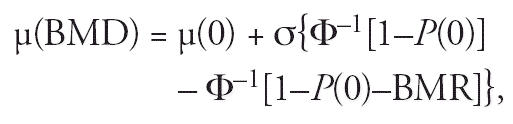


where Φ^–1^ is the inverse of the standard normal cumulative distribution function and *P*(0) is the cutoff level for adverse response defined in terms of a specified tail proportion of a “hypothetical” control distribution (at U-Cd = 0), equivalent to the background probability of adverse response. The cutoff, *c*, for the effect markers is given by


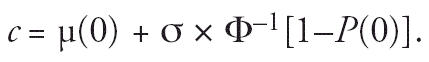


The BMD is obtained by combining the equation for μ(BMD) with that for the dose–response (Equation 1):


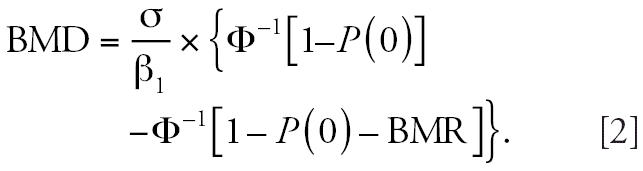


For negative associations between exposure and effects (β_1_ < 0), such as that for the association between Cd and estimated GFR, calculations were performed in a similar way, substituting the absolute value of β_1_ into Equation 2 (Sand et al. 2003).

The BMDL was calculated using the profile likelihood method (Crump 1995; Filipsson et al. 2003). BMDs/BMDLs with the *P*(0) = 5% and BMR = 5 or 10% were calculated for all renal effect markers as representative threshold levels. To describe how the BMD/BMDL depends on the BMR and *P*(0), data on NAG adjusted for specific gravity were used as an example. We calculated BMD/BMDL for three different BMRs (5, 10, or 20%) with varying *P*(0) (1–20%).

### Comparisons with previously used procedures

To compare the hybrid approach with the previously used procedures, in which the outcome is dichotomized, we applied the procedures used in the Cadmibel study (Buchet et al. 1990) and the OSteoporosis, CAdmium as a Risk Factor (OSCAR) study (Järup et al. 2000) on our data. In the Cadmibel study, the relationship between renal adverse response and U-Cd was investigated in a Belgian population (Buchet et al. 1990). The authors derived the cutoffs as the 95th percentile of the renal tubular markers in a part of the study population that was considered to be free from kidney disease. The U-Cd levels at which the probability of having an adverse response was 10% were estimated using a logistic model after exclusion of individuals with diabetes and regular use of NSAIDs. In the OSCAR study (Järup et al. 2000), the adverse response of protein HC in relation to U-Cd was investigated in a Swedish population. The adverse response was defined as urinary protein HC above the 95th percentile (5.3 mg/g creatinine; 0.6 mg/mmol creatinine in women) from another Swedish reference population (Tencer et al. 1996). The U-Cd level at 15% probability of an adverse response was then estimated using parameters obtained by logistic regression model in the OSCAR study.

We fitted our data to a logistic model. The probability of adverse response at the dose *d_i_* of U-Cd is given by


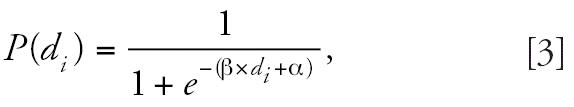


where αis the log odds of adverse response at the U-Cd = 0, and βis the slope for dose–log odds relationship. Then, d*_i_* is given by


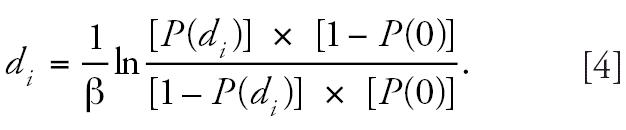


The background probabilities and the U-Cd levels at the 10% (Cadmibel) or 15% (OSCAR) probability of adverse response were estimated by Equations 3 and 4 and compared with corresponding background probability and U-Cd levels using the hybrid approach.

### Software

We used SPSS (version 12.0.1; SPSS Inc., Chicago, IL, USA) and Microsoft Excel (Microsoft Corp., Redmond, WA, USA) for analyses. These results were verified to be identical to the results by MATLAB (version 7.0; MathWorks, Inc., Novi, MI, USA) used in our previous studies (Sand et al. 2003, 2004).

## Results

We found significant associations between U-Cd, on the one hand, and NAG, protein HC (both positive associations), and estimated GFR (negative association), on the other, based on the maximum likelihood model (data not shown).

Table 2 shows the BMDs and BMDLs of U-Cd using a cutoff, *P*(0), of 5% and a BMR of 5 or 10% for the renal effect markers. For the tubular effects (both NAG and protein HC), the BMDLs of U-Cd were 0.4–0.8 μg/L, corresponding to 0.5–0.8 μg/g creatinine. For the glomerular effects (estimated GFR), the BMDLs of U-Cd were 0.5–0.9 μg/L, corresponding to 0.7–1.2 μg/g creatinine. We obtained essentially the same BMD/BMDL if we used cystatin C instead of estimated GFR.

**Figure 1:**
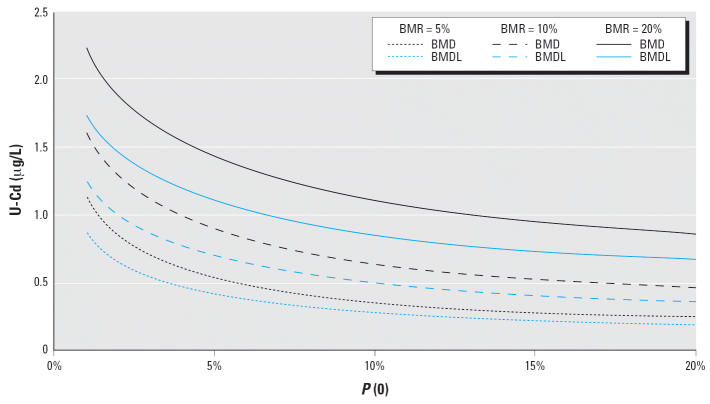
BMDs with BMDLs for U-Cd based on the tubular marker NAG (U/L) in relation to different cutoff levels [*P*(0)] and BMRs. Both markers were adjusted to a specific gravity of 1.015 g/mL.

We evaluated the effect of the cutoff value [*P*(0)] and the response criteria (BMR) on the BMDs/BMDLs. As shown in Figure 1, a larger BMR and a smaller *P*(0) yield larger BMD/BMDLs.

**Figure 2:**
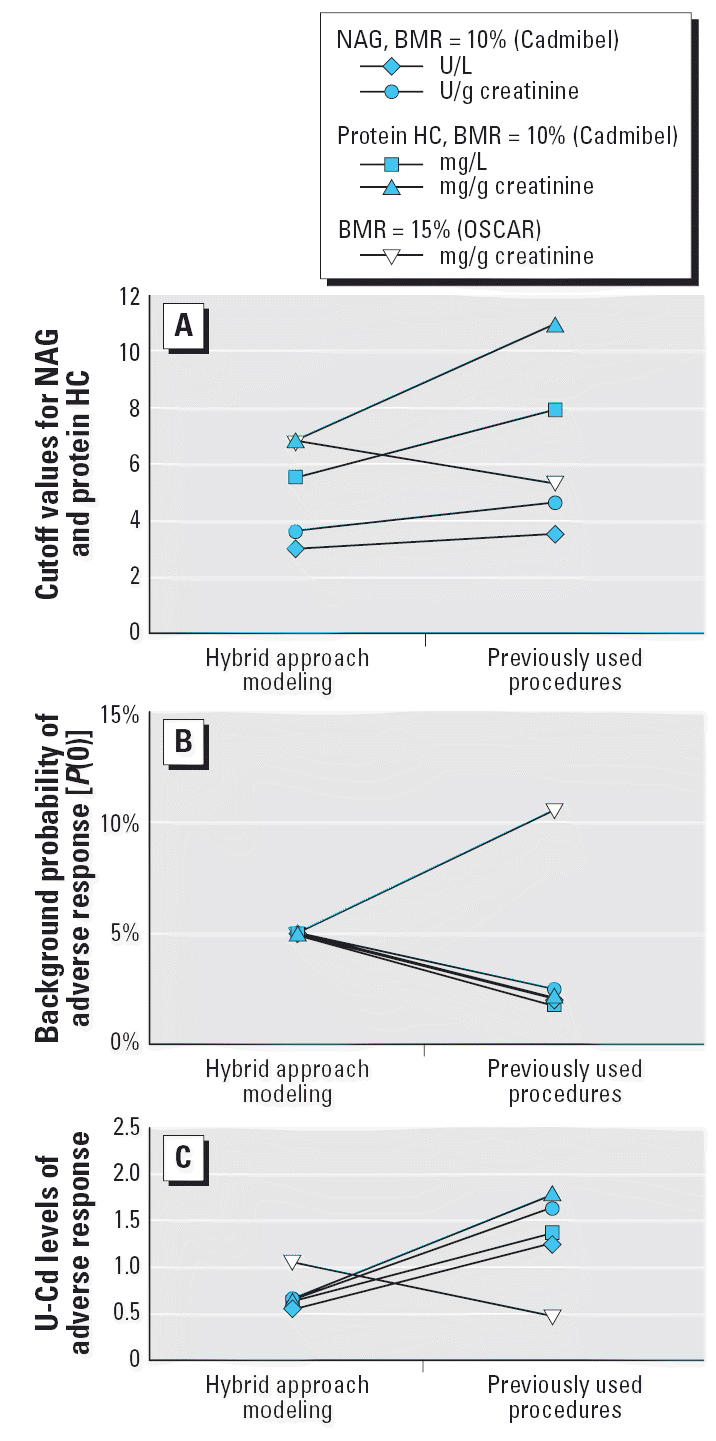
Cutoff values for the tubular markers (*A*), corresponding background probabilities of adverse response (*B*), and U-Cd at predetermined probabilities of adverse response (*C*) by applying previously used procedures and hybrid approach modeling to our data. The values presented for the hybrid approach correspond to the BMDs shown in Table 2.

As shown in Figure 2A, the cutoff concentrations of NAG and protein HC obtained by our hybrid approach modeling were lower than those obtained by employing the procedure used in the Cadmibel study in our study. The opposite was observed for the OSCAR procedure. In Figure 2B, the cutoffs from Figure 2A are presented in terms of different background probabilities of adverse response [*P*(0)]. By using the predefined cutoff values of the Cadmibel and OSCAR studies, we obtained a lower and a higher *P*(0), respectively (Figure 2B). When we compared the critical concentration of U-Cd obtained by the hybrid approach, the U-Cd levels were lower than those obtained by applying the Cadmibel procedure to our data. The opposite was observed for the OSCAR procedure (Figure 2C).

## Discussion

To our knowledge, this is the first estimation of BMDs of Cd-induced renal effects using the recently developed hybrid approach. The critical concentration was estimated for both tubular and glomerular effects in a population of upper middle-age women living in an area in southern Sweden without particular industrial Cd emission. Generally, the critical concentrations obtained by the hybrid method approach were lower than those previously reported.

The present method has several methodologic advantages. First, the BMDs/BMDLs were calculated based on a continuous outcome. Calculations of BMD/BMDL for continuous outcomes using the hybrid approach has been developed during the last few years (Crump 1995; Sand et al. 2004). The advantage with the hybrid approach is that the categorization of subjects with respect to the outcome variables can be avoided. Accordingly, the statistical validity and efficiency of the BMD is higher using the hybrid approach, compared with methods involving dichotomization of a continuous outcome (Crump 2002; West and Kodell 1999).

Second, we defined the cutoff for adverse effects as the 95th percentile, obtained by the model at no Cd exposure (U-Cd = 0) in the population under study, rather than as the 95th percentile of the effect marker in an apparently low-exposed “reference” population, with little information on the overall comparability. Further, by estimating the cutoff for adverse response by the model at zero Cd exposure, any impact of the exposure level in a reference group will be minimized. The obtained critical U-Cd levels then corresponds to an adverse response of 10% (5% additional probability of adverse response; BMR = 5%) or 15% (10% additional probability of adverse response; BMR = 10%).

Third, we were able to avoid categorization of the exposure variable. Except for the fact that the number categories and the dose interval for each category chosen may strongly affect the result, categorization will decrease the detection power (Royston et al. 2000).

Furthermore, we further improved the method by using a multivariate linear regression model instead of a univariate model (Hong et al. 2004; Jin et al. 2004; Uno et al. 2005) to enable the adjustment of BMD/ BMDL for potential confounders.

The BMDs for U-Cd obtained in the present study were generally lower than the previously reported critical levels. For instance, the lowest observed effect levels based on the same data (Åkesson et al. 2005) were on average 10% higher than the present BMDs. In the Cadmibel study (Buchet et al. 1990), the U-Cd level corresponding to the 10% probability of adverse response was 1.9 μg/24 hr (equivalent to about 2 μg/g creatinine) for calciuria and 2.7 μg/24 hr (roughly equivalent to 3 μg/g creatinine) for NAG. However, in the OSCAR study (Järup et al. 2000), the U-Cd level corresponding to 15% probability of adverse response was 1.0 μg/g creatinine, similar to that obtained in the present study. Further, the BMDLs obtained in the present study were clearly lower than the BMDLs of 4–12 μg Cd/g creatinine (5% additional probability) obtained for various kidney effect markers (NAG and isoform B), β_2_-microglobulin (β_2_-MG), retinol-binding protein, and urinary albumin in China (Jin et al. 2004), and slightly lower than the 0.9–1.2 μg Cd/g creatinine (10% additional probability) (Hong et al. 2004) obtained in another Chinese population that was coexposed to arsenic. The present BMDLs were, however, very similar to that obtained in Japanese women 40–59 years of age in a Cd-nonpolluted area. The BMDLs (5% additional probability) for the kidney effects (total protein, β_2_-MG, and NAG) were 0.6–1.8 μg/g creatinine (Uno et al. 2005). The corresponding results for men were lower: 0.3–0.6 μg/g creatinine.

All of these other studies defined the adverse response (cutoff) as the 95th percentile in a reference population assumed to be non-exposed (Hong et al. 2004; Järup et al. 2000; Jin et al. 2004) or in a part of the study population considered free from kidney disease (Buchet et al. 1990; Uno et al. 2005). The study subjects were then categorized (dichotomous) as to the outcome. Obviously, a more Cd-exposed reference group (Jin et al. 2002) showed a considerably higher critical concentration (Jin et al. 2004), emphasizing the need for a better standardized method to obtain the threshold for adverse response. In addition, all the previous studies on U-Cd and BMD (Hong et al. 2004; Jin et al. 2004; Uno et al. 2005) categorized the exposure into strata (Benchmark Dose Software; U.S. EPA, Washington, DC, USA). We consider the present continuous approach of calculating the BMD/BMDL more accurate and more efficient in using the information.

When we applied previously used methods to our data, the procedure used in the Cadmibel study for defining the cutoff resulted in a background probability of < 5%, whereas the procedure used in the OSCAR study resulted in a background probability of > 5%. The main reason for the higher *P*(0) in the latter study was that the reference population was, on average, 20 years younger than the study population. As illustrated in Equation 4 for the logistic regression, a low background probability of adverse response *P*(0) yields a larger [1 – *P*(0)]/*P*(0), which may yield a larger critical U-Cd level (for a constantβ). Considered together, this shows that the cutoff value has a strong effect on the estimated critical level. Thus, the choice of reference population for determination of the cutoff for adverse effects (95th percentile) may have large impact on the critical concentrations. The advantage of the hybrid approach is that it allows for estimation of the cutoff at zero exposure in the population under study.

Although, compared with other methods, the hybrid approach seemed to be better in terms of the obtained critical concentration, it is still, as for the logistic model, influenced by the actual value of the background probability of adverse response, *P*(0). On the other hand, by using the hybrid approach, it is always possible to set a defined *P*(0), which allows for interpopulation comparison of critical concentrations under the same conditions. This is important because a lower *P*(0) leads to larger BMD/BMDL, as shown in Figure 1. The reason for this relates to the characteristics of the normal distribution. The absolute distance between two points on the distribution axis that bracket, for example, a 5% probability (i.e., a BMR = 5%) becomes higher in the extreme tail region compared with in a more central part of the distribution. Thus, the lower *P*(0) becomes, the greater the distance between the two points corresponding to *P*(0) and *P*(0) + BMR, which translates to a higher dose (BMD) being required to produce the desired change in probability (BMR). Furthermore, the impact of *P*(0) on BMD was more pronounced at lower than at higher values of *P*(0). To our knowledge, such importance of background probability has not previously been evaluated in detail for either the hybrid approach or the logistic model.

In several previous studies, a *P*(0) of 5% has been used as a standard for the hybrid approach (Budtz-Jørgensen et al. 2000; Crump et al. 2000; Jacobson et al. 2002; Murata et al. 2002, 2004), in accordance with the usual definition of clinical reference intervals. The adopted BMR levels in the present study are in line with those used in other recent epidemio-logic studies: 5% (Budtz-Jørgensen et al. 2000; Murata et al. 2002, 2004) or 10% (Budtz-Jørgensen et al. 2000; Crump et al. 2000). Obviously, other *P*(0) values and BMRs can be chosen, depending on the severity of the effects (Jacobson et al. 2002).

The population-based design and the rather high participation rate advocate generalization of the results to other female populations in the same age interval. However, we cannot exclude gender difference in BMDLs for kidney effects (Uno et al. 2005).

In conclusion, the present BMDLs for tubular effects, using a cutoff *P*(0) of 5%, were 0.4 μg/L (0.5 μg/g creatinine) at a BMR of 5% and 0.7 μg/L (0.8 μg/g creatinine) using a BMR of 10%. The corresponding BMDLs for the glomerular effect were 0.5 μg/L (0.7 μg/g creatinine) and 0.9 μg/L (1.2 μg/g creatinine) for BMRs of 5 and 10%, respectively. This critical U-Cd level for glomerular effects was lower and closer to the critical levels for tubular effects than expected from previous studies.

## Figures and Tables

**Table 1:** Exposure and effect markers.

Effect marker	No.	Mean ± SD
U-Cd		
μg/L	790	0.61 ± 0.36
μg/g creatinine		0.76 ± 0.42
NAG		
U/L	790	1.42 ± 1.09
U/g creatinine		1.78 ± 1.43
Protein HC		
mg/L	790	3.05 ± 2.38
mg/g creatinine		3.92 ± 3.19
Serum cystatin C (mg/L)	700	0.82 ± 0.13
Estimated GFR (mL/min)	700	102.2 ± 18.9

**Table 2:** BMDs with their lower bounds (BMDL) corresponding to 5 and 10% additional risk (BMR) calculated using the hybrid approach.

		U-Cd BMD (BMDL)	
Effect marker	Cutoff^a^	5% BMR	10% BMR
NAG			
U/L	3.0	0.53 (0.41 μg/L)^b^	0.89 (0.69 μg/L)^b^
U/g creatinine	3.6	0.64 (0.50 μg/g creatinine)	1.08 (0.83 μg/g creatinine)
Protein HC			
mg/L	5.5	0.63 (0.47 μg/L)^b^	1.05 (0.78 μg/L)^b^
mg/g creatinine	6.8	0.63 (0.49 μg/g creatinine)	1.05 (0.81 μg/g creatinine)
Estimated GFR			
mL/min	82.6	0.80 (0.55 μg/L)^b^	1.34 (0.92 μg/L)^b^
mL/min	78.5	1.08 (0.70 μg/g creatinine)	1.80 (1.18 μg/g creatinine)

a Cutoff values were defined as 95th percentile of effect markers on the “hypothetical” control distrubution at U-Cd = 0.

b U-Cd was adjusted to mean specific gravity of 1.015.
